# Supplementary material for: A Scoping Review of Climate Change, Climate-Related Disasters, and Mental Disorders among Children in Low- and Middle-Income Countries
Source: Int J Environ Res Public Health. 2022 Mar 2;19(5):2896. doi: 10.3390/ijerph19052896 (PMC8910112; doi:10.3390/ijerph19052896)
Supplement: Supplementary file 1 [file ijerph-19-02896-s001.zip › ijerph-1544533-supplementary.pdf]

Supplementary Materials

Table S1: Inclusion and exclusion criteria for the climate-related disaster exposures. The original list of disasters came from the International Disaster Database (EM-DAT) (https://www.emdat.be/). We determined whether a disaster was climate-related based on previous categorizations in the literature [1,2].

Included (climate-related)	Excluded (not climate-related)
**Extreme temperature** (cold wave, heat wave, severe winter conditions such as snow/ice or frost/freeze)	**Earthquake** (ground movement, tsunami)
**Fog**	**Dry mass movement** (rockfall, landslide)
**Storm** (extra-tropical storms, tropical storms, convective storms – includes derecho, hail, lightning/thunderstorm, rain, tornado, sand/dust storm, storm/surge, wind)	**Volcanic activity** (ash fall, lahar, pyroclastic flow, lava flow)
**Flood** (coastal flood, riverine flood, flash flood, ice jam flood)	**Epidemic** (viral, bacterial, parasitic, fungal, prion)
**Landslide** (avalanche – snow debris, mudflow, rockfall) ***not included if it is a dry rockfall or landslide*	**Insect infestation** (grasshopper, locust)
**Wave action** (rogue wave, seiche) ***not included if it is a tsunami triggered by an earthquake*	**Animal accident**
**Drought**	**Extraterrestrial impact** (airburst)
**Glacial lake outburst**	**Space weather** (energetic particles, geomagnetic storm, shockwave)
**Wildfire** (forest fire, land fire – including brush, bush, and pasture fires)	**Any technological/human-made disaster** (e.g., chemical spill, explosion, collapse, fire, gas leak, oil spill, transport accident)

Table S2: Detailed MEDLINE search strategy.

Line	Search Terms
1	exp Climate change/
2	exp Climate/
3	Natural disasters/
4	Avalanches/
5	Cyclonic storms/
6	Droughts/
7	Floods/
8	Landslides/
9	Tornadoes/
10	Wildfires/
11	Disasters/
12	Weather/
13	Extreme weather/
14	Wind/
15	Humidity/
16	Rain/
17	Snow/
18	exp Temperature/
19	Lightning/
20	Ice/
21	Greenhouse effect/
22	Greenhouse gases/
23	exp Biodiversity/
24	exp Particulate matter/
25	Air pollution/
26	Air pollutants/
27	Water quality/
28	Water pollutants/

29	Soil pollutants/
30	("climate change*" or "climatic change*" or disaster* or "weather event*" or "extreme weather" or "global warming" or "climatic variability" or "air quality" or "water quality" or "pollution" or "land use" or "ecological change*" or "greenhouse gas*" or "extreme temperature*" or "cold wave*" or "heat wave*" or snow or snows or snowstorm* or ice or frost* or freez* or fog* or storm* or derecho* or hail* or thunderstorm* or lightning or rain* or tornado* or wind* or flood* or landslide* or avalanche* or rockfall* or mudflow* or "wave action*" or "rogue wave*" or seiche* or drought* or "glacial lake outburst*" or wildfire* or "forest fire*" or "land fire*" or "brush fire*" or "bush fire*" or "pasture fire*").mp.
31[a]	(**afghanistan** OR **albania** OR **algeria** OR **american samoa** OR **angola** OR **"antigua and barbuda"** OR antigua OR barbuda OR **argentina** OR **armenia** OR armenian OR **aruba** OR **azerbaijan** OR **bahrain** OR **bangladesh** OR **barbados** OR **republic of belarus** OR belarus OR byelarus OR belorussia OR byelorussian OR **belize** OR british honduras OR **benin** OR dahomey OR **bhutan** OR **bolivia** OR **"bosnia and herzegovina"** OR bosnia OR herzegovina OR **botswana** OR bechuanaland OR **brazil** OR brasil OR **bulgaria** OR **burkina faso** OR burkina fasso OR upper volta OR **burundi** OR urundi OR **cabo verde** OR cape verde OR **cambodia** OR kampuchea OR khmer republic OR **cameroon** OR cameron OR cameroun OR **central african republic** OR ubangi shari OR **chad** OR **chile** OR **china** OR **colombia** OR **comoros** OR comoro islands OR iles comores OR mayotte OR **democratic republic of the congo** OR democratic republic congo OR **congo** OR zaire OR **costa rica** OR **"cote d'ivoire"** OR "cote d' ivoire" OR cote divoire OR cote d ivoire OR ivory coast OR **croatia** OR **cuba** OR **cyprus** OR **czech republic** OR czechoslovakia OR **djibouti** OR french somaliland OR **dominica** OR **dominican republic** OR **ecuador** OR **egypt** OR united arab republic OR **el salvador** OR **equatorial guinea** OR spanish guinea OR **eritrea** OR **estonia** OR eswatini OR **swaziland** OR **ethiopia** OR **fiji** OR **gabon** OR gabonese republic OR **gambia** OR **"georgia (republic)"** OR georgian OR **ghana** OR gold coast OR **gibraltar** OR **greece** OR **grenada** OR **guam** OR **guatemala** OR **guinea** OR **guinea bissau** OR **guyana** OR british guiana OR **haiti** OR hispaniola OR **honduras** OR **hungary** OR **india** OR **indonesia** OR timor OR **iran** OR **iraq** OR isle of man OR **jamaica** OR **jordan** OR **kazakhstan** OR kazakh OR **kenya** OR "**democratic people's republic of korea"** OR **republic of korea** OR north korea OR south korea OR korea OR **kosovo** OR **kyrgyzstan** OR kirghizia OR kirgizstan OR kyrgyz republic OR kirghiz OR **laos** OR lao pdr OR "lao people's democratic republic" OR **latvia** OR **lebanon** OR lebanese republic OR **lesotho** OR basutoland OR **liberia** OR **libya** OR libyan arab jamahiriya OR **lithuania** OR **macau** OR macao OR **"macedonia (republic)"** OR macedonia OR **madagascar** OR malagasy republic OR **malawi** OR nyasaland OR **malaysia** OR malay federation OR malaya federation OR maldives OR **indian ocean islands** OR indian ocean OR **mali** OR **malta** OR **micronesia** OR federated states of micronesia OR kiribati OR marshall islands OR nauru OR northern mariana islands OR **palau** OR tuvalu OR **mauritania** OR **mauritius** OR **mexico** OR **moldova** OR moldovian OR **mongolia** OR **montenegro** OR **morocco** OR ifni OR **mozambique** OR portuguese east africa OR **myanmar** OR burma OR **namibia** OR **nepal** OR **netherlands antilles** OR **nicaragua** OR **niger** OR **nigeria** OR **oman** OR muscat OR **pakistan** OR **panama** OR **papua new guinea** OR new guinea OR **paraguay** OR **peru** OR **philippines** OR philipines OR phillipines OR phillippines OR **poland** OR "polish people's republic" OR **portugal** OR portuguese republic OR **puerto rico** OR **romania** OR **russia** OR russian federation OR ussr OR soviet union OR union of soviet socialist republics OR **rwanda** OR ruanda OR **samoa** OR pacific islands OR polynesia OR samoan islands OR navigator island OR navigator islands OR **"sao tome and principe"** OR **saudi arabia** OR **senegal** OR **serbia** OR **seychelles** OR **sierra leone** OR **slovakia** OR slovak republic OR **slovenia** OR **melanesia** OR solomon island OR solomon islands OR norfolk island OR norfolk islands OR **somalia** OR **south africa** OR **south sudan** OR **sri lanka** OR ceylon OR **"saint kitts and nevis"** OR "st. kitts and nevis" OR **saint lucia** OR "st. lucia" OR **"saint vincent and the grenadines"** OR saint vincent OR "st. vincent" OR grenadines OR **sudan** OR **suriname** OR surinam OR dutch guiana OR netherlands guiana OR **syria** OR syrian arab republic OR **tajikistan** OR tadjikistan OR tadzhikistan OR tadzhik OR **tanzania** OR tanganyika OR **thailand** OR siam OR **timor leste** OR east timor OR **togo** OR togolese republic OR **tonga** OR **"trinidad and tobago"** OR trinidad OR tobago OR **tunisia** OR **turkey** OR "turkey (republic)" OR **turkmenistan** OR turkmen OR **uganda** OR **ukraine** OR **uruguay** OR **uzbekistan** OR uzbek OR **vanuatu** OR new hebrides OR **venezuela** OR **vietnam** OR viet nam OR **middle east** OR west bank OR gaza OR palestine OR **yemen** OR **yugoslavia** OR **zambia** OR **zimbabwe** OR northern rhodesia OR global south OR **africa south of the sahara** OR sub-saharan africa OR subsaharan africa OR **africa, central** OR central africa OR **africa, northern** OR north africa OR northern africa OR magreb OR maghrib OR sahara OR **africa, southern** OR southern africa OR **africa, eastern** OR east africa OR eastern africa OR **africa, western** OR west africa OR western africa OR **west indies** OR **indian ocean islands** OR **caribbean** OR **central america** OR **latin america** OR "south and central america" OR **south america** OR **asia, central** OR central asia OR **asia, northern** OR north asia OR northern asia OR **asia, southeastern** OR southeastern asia OR south eastern asia OR southeast asia OR south east asia OR **asia, western** OR western asia OR **europe, eastern** OR east europe OR eastern europe OR developing country OR **developing countries** OR developing nation? OR developing population? OR developing world OR less developed countr* OR less developed nation? OR less developed population? OR less developed world OR lesser developed countr* OR lesser developed nation? OR lesser developed population? OR lesser developed world OR under developed countr* OR under developed nation? OR under developed population? OR under developed world OR underdeveloped countr* OR underdeveloped nation? OR underdeveloped population? OR underdeveloped world OR middle income countr* OR middle income nation? OR middle income population? OR low income countr* OR low income nation? OR low income population? OR lower income countr* OR lower income nation? OR lower income population? OR underserved countr* OR underserved nation? OR underserved population? OR underserved world OR under served countr* OR under served nation? OR under served population? OR under served world OR deprived countr* OR deprived nation? OR deprived population? OR deprived world OR poor countr* OR poor nation? OR poor population? OR poor world OR poorer countr* OR poorer nation? OR poorer population? OR poorer world OR developing econom* OR less developed econom* OR lesser developed econom* OR under developed econom* OR underdeveloped econom* OR middle income econom* OR low income econom* OR lower income econom* OR low gdp OR low gnp OR low gross domestic OR low gross national OR lower gdp OR lower gnp OR lower gross domestic OR lower gross national OR lmic OR lmics OR third world OR lami countr* OR transitional countr* OR emerging economies OR emerging nation?**)**.ti,ab,sh,kf.
32[a]	(afghan OR afghans OR afghani OR albanian? algerian? OR american samoan? OR angolan? OR antiguan? OR barbudan? OR argentine? OR argentinian? OR argentinean? OR armenian? OR aruban? OR azerbaijani? OR bahraini? OR bangladeshi? OR bangalees OR bajan? OR belarusian? OR byelorussian? OR belizean? OR beninese? OR bhutanese OR bolivian? OR bosnian? OR botswana OR batswana OR brazilian? OR brasilian? OR bulgarian? OR burkinabe OR burkinese OR burundian? OR cape verdean? OR cabo verdean? OR cambodian? OR khmer OR cameroonian? OR central african? OR chadian? OR chilean? OR chinese OR colombian? OR comorian? OR congolese OR costa rican? OR ivorian? OR croatian? OR cuban? OR cypriot? OR czech? OR djiboutian? OR dominican? OR ecuadorian? OR egyptian? OR salvadoran? OR equatorial guinean? OR equatoguinean? OR eritrean? OR estonian? OR swazi? OR swati? OR ethiopian? OR fijian OR gabonese OR gabonaise OR gambian? OR georgian? OR ghanaian? OR gibraltarian? OR greek? OR grenadian? OR guamanian? OR guatemalan? OR guinean? OR bissau guinean? OR guyanese OR haitian? OR honduran? OR hungarian? OR indian? OR indonesian? OR iranian? OR iraqian? OR iraqi? OR manx OR jamaican? OR jordanian? OR kazakhstani? OR kenyan? OR kirabati OR kirabatian? OR north korean? OR korean? OR kosovar? OR kosovan? OR kyrgyz* OR lao OR laotian? OR latvian? OR lebanese OR lesothan? OR lesothonian? OR mosotho OR basotho OR liberian? OR libyan? OR lithuanian? OR macanese OR macedonian? OR malagasy OR madagascan? OR malawian? OR malaysian? OR maldivian? OR malian? OR maltese OR marshallese? OR mauritanian? OR mauritian? OR mexican? OR micronesian? OR moldovan? OR mongolian? OR mongol OR montenegrin? OR moroccan? OR mozambican? OR burmese OR myanma OR namibian? OR nauruan? OR nepali OR nepalese OR netherlands antillean? OR nicaraguan? OR nigerien? OR nigerian? OR northern mariana islander? OR mariana? OR omani? OR pakistani? OR

	palauan? OR panamanian? OR papua new guinean? OR paraguayan? OR peruvian? OR philippine? OR philipine? OR phillipine? OR phillippine? OR filipino? OR filipina? OR polish OR pole OR poles OR portuguese OR puerto rican? OR romanian? OR russian? OR soviet people OR soviet population OR rwandan? OR rwandese OR ruandan? OR ruandese OR samoan? OR sao tomean? OR santomean? OR saudi arabian? OR saudi? OR senegalese OR serbian? OR montenegrin? OR seychellois OR seychelloise? OR sierra leonean? OR slovak? OR slovene? OR solomon islander? OR somali? OR south african? OR south sudanese OR sri lankan? OR ceylonese OR kittitian? OR nevisian? OR saint lucian? OR vincentian? OR sudanese OR surinamese? OR syrian? OR tajik? OR tajikistani? OR tanzanian? OR tanganyikan? OR thai OR timorese? OR togolese OR tongan? OR trinidadian? OR tobagonian? OR tunisian? OR turk? OR turkish OR turkmen? OR tuvaluan? OR ugandan? OR ukrainian? OR uruguayan? OR uzbek? OR vanuatu* OR venezuelan? OR vietnamese OR yemeni? OR yemenite? OR yemenese OR yugoslav? OR yugoslavian? OR zambian? OR zimbabwean?).ti,ab,sh,kf.
33	Mental health/
34	Mental disorders/
35	exp Anxiety disorders/
36	Anxiety/
37	exp Mood disorders/
38	Depression/
39	exp Substance-related disorders/
40	exp Prescription drug misuse/
41	exp Stress disorders, traumatic
42	exp Suicide
43	Self-injurious behaviour/
44	("mental health" or "mental illness*" or "mental disorder*" or "psychological disorder*" or "psychiatric disorder*" or psychopatholog* or anxiet* or depress* or "post-traumatic stress" or "posttraumatic stress" or PTSD or suicide or "self harm" or "substance use" or "substance abuse" or "alcohol use" or "alcohol abuse" or "drug use" or "drug abuse" or "mood disorder*").mp.
45	1 or 2 or 3 or 4 or 5 or 6 or 7 or 8 or 9 or 10 or 11 or 12 or 13 or 14 or 15 or 16 or 17 or 18 or 19 or 20 or 21 or 22 or 23 or 24 or 25 or 26 or 27 or 28 or 29 or 30
46	31 or 32
47	33 or 34 or 35 or 36 or 37 or 38 or 39 or 40 or 41 or 42 or 43 or 44
48	45 and 46 and 47
49	48 not ((exp animals/ or exp invertebrates/ or animal experiments/ or animal models/ or exp plants/ or exp fungi/) not exp humans/)
50	Limit 49 to yr="2007 – 2019"
**TOTAL RESULTS: 2,547**	

[a]Used Cochrane's LMIC filters (https://epoc.cochrane.org/lmic-filters). Bolded terms represent subject headings.

Table S3: Detailed EMBASE search strategy.

Line	Search Terms
1	Climate resilience/
2	exp Environmental impact/
3	exp Climate/
4	Natural disaster/
5	Disaster/
6	Avalanche/
7	Storm surge/
8	exp Tide/
9	exp Season/
10	Greenhouse gas/
11	Smog/
12	Photochemical smog/
13	exp Weather/
14	Fire/
15	exp Wildfire/
16	Biosphere/
17	Habitat/
18	Air pollution/
19	Air quality/
20	Particulate matter/
21	Air pollutant/
22	Water quality/
23	Water pollution/
24	Water pollutant/
25	Soil pollution/
26	Soil pollutant/
27	("climate change*" or "climatic change*" or disaster* or "weather event*" or "extreme weather" or "global warming" or "climatic variability" or "air quality" or "water quality" or "pollution" or "land use" or "ecological change*" or "greenhouse gas*" or "extreme temperature*" or "cold wave*" or "heat wave*" or snow or snows or snowstorm* or ice or frost* or freez* or fog* or storm* or derecho* or hail* or thunderstorm* or lightning or rain* or tornado* or wind* or flood* or landslide* or avalanche* or rockfall* or mudflow* or "wave action*" or "rogue wave*" or

	seiche* or drought* or "glacial lake outburst*" or wildfire* or "forest fire*" or "land fire*" or "brush fire*" or "bush fire*" or "pasture fire*").mp.
28[a]	(**afghanistan** OR **albania** OR **algeria** OR **american samoa** OR **angola** OR **"antigua and barbuda"** OR antigua OR barbuda OR **argentina** OR **armenia** OR armenian OR **aruba** OR **azerbaijan** OR **bahrain** OR **bangladesh** OR **barbados** OR republic of belarus OR **belarus** OR byelarus OR belorussia OR byelorussian OR **belize** OR british honduras OR **benin** OR dahomey OR **bhutan** OR **bolivia** OR **"bosnia and herzegovina"** OR bosnia OR herzegovina OR **botswana** OR bechuanaland OR **brazil** OR brasil OR **bulgaria** OR **burkina faso** OR burkina fasso OR upper volta OR **burundi** OR urundi OR cabo verde OR **cape verde** OR **cambodia** OR kampuchea OR khmer republic OR **cameroon** OR cameron OR cameroun OR **central african republic** OR ubangi shari OR **chad** OR **chile** OR **china** OR **colombia** OR **comoros** OR comoro islands OR iles comores OR mayotte OR democratic republic of the congo OR **democratic republic congo** OR **congo** OR zaire OR **costa rica** OR **"cote d'ivoire"** OR "cote d' ivoire" OR cote divoire OR cote d ivoire OR ivory coast OR **croatia** OR **cuba** OR **cyprus** OR **czech republic** OR czechoslovakia OR **djibouti** OR french somaliland OR **dominica** OR **dominican republic** OR **ecuador** OR **egypt** OR united arab republic OR **el salvador** OR **equatorial guinea** OR spanish guinea OR **eritrea** OR **estonia** OR eswatini OR **swaziland** OR **ethiopia** OR **fiji** OR **gabon** OR gabonese republic OR **gambia** OR **"georgia (republic)"** OR georgian OR **ghana** OR gold coast OR **gibraltar** OR **greece** OR **grenada** OR **guam** OR **guatemala** OR **guinea** OR **guinea bissau** OR **guyana** OR british guiana OR **haiti** OR hispaniola OR **honduras** OR **hungary** OR **india** OR **indonesia** OR timor OR **iran** OR **iraq** OR **isle of man** OR **jamaica** OR **jordan** OR **kazakhstan** OR kazakh OR **kenya** OR "democratic people's republic of korea" OR republic of korea OR **north korea** OR **south korea** OR korea OR **kosovo** OR **kyrgyzstan** OR kirghizia OR kirgizstan OR kyrgyz republic OR kirghiz OR **laos** OR lao pdr OR "lao people's democratic republic" OR **latvia** OR **lebanon** OR lebanese republic OR **lesotho** OR basutoland OR **liberia** OR libya OR **libyan arab jamahiriya** OR **lithuania** OR **macau** OR macao OR **"macedonia (republic)"** OR macedonia OR **madagascar** OR malagasy republic OR **malawi** OR nyasaland OR **malaysia** OR malay federation OR malaya federation OR maldives OR indian ocean islands OR **indian ocean** OR **mali** OR **malta** OR micronesia OR **federated states of micronesia** OR **kiribati** OR marshall islands OR nauru OR northern mariana islands OR palau OR tuvalu OR **mauritania** OR **mauritius** OR **mexico** OR **moldova** OR moldovian OR **mongolia** OR montenegro OR **"montenegro (republic)"** OR **morocco** OR ifni OR **mozambique** OR portuguese east africa OR **myanmar** OR burma OR **namibia** OR **nepal** OR **netherlands antilles** OR **nicaragua** OR **niger** OR **nigeria** OR **oman** OR muscat OR **pakistan** OR **panama** OR **papua new guinea** OR new guinea OR **paraguay** OR **peru** OR **philippines** OR philipines OR phillipines OR phillippines OR **poland** OR "polish people's republic" OR **portugal** OR portuguese republic OR **puerto rico** OR **romania** OR russia OR **russian federation** OR ussr OR soviet union OR union of soviet socialist republics OR **rwanda** OR ruanda OR **samoa** OR pacific islands OR polynesia OR samoan islands OR navigator island OR navigator islands OR **"sao tome and principe"** OR **saudi arabia** OR **senegal** OR **serbia** OR **seychelles** OR **sierra leone** OR **slovakia** OR slovak republic OR **slovenia** OR **melanesia** OR solomon island OR solomon islands OR norfolk island OR norfolk islands OR **somalia** OR **south africa** OR **south sudan** OR **sri lanka** OR ceylon OR **"saint kitts and nevis"** OR "st. kitts and nevis" OR **saint lucia** OR "st. lucia" OR **"saint vincent and the grenadines"** OR saint vincent OR "st. vincent" OR grenadines OR **sudan** OR **suriname** OR surinam OR dutch guiana OR netherlands guiana OR syria OR **syrian arab republic** OR **tajikistan** OR tadjikistan OR tadzhikistan OR tadzhik OR **tanzania** OR tanganyika OR **thailand** OR siam OR **timor leste** OR east timor OR **togo** OR togolese republic OR **tonga** OR **"trinidad and tobago"** OR trinidad OR tobago OR **tunisia** OR turkey OR **"turkey (republic)"** OR **turkmenistan** OR turkmen OR **uganda** OR **ukraine** OR **uruguay** OR **uzbekistan** OR uzbek OR **vanuatu** OR new hebrides OR **venezuela** OR vietnam OR **viet nam** OR middle east OR west bank OR gaza OR **palestine** OR **yemen** OR **yugoslavia** OR **zambia** OR **zimbabwe** OR northern rhodesia OR global south OR **africa south of the sahara** OR "sub saharan africa" OR subsaharan africa OR **africa, central** OR central africa OR **africa, northern** OR north africa OR northern africa OR magreb OR maghrib OR sahara OR **africa, southern** OR southern africa OR **africa, eastern** OR east africa OR eastern africa OR **africa, western** OR west africa OR western africa OR **west indies** OR **indian ocean islands** OR **caribbean region** OR caribbean islands OR caribbean OR **central america** OR latin america OR "south and central america" OR **south america** OR **asia, central** OR central asia OR **asia, northern** OR north asia OR northern asia OR **asia, southeastern** OR southeastern asia OR south eastern asia OR southeast asia OR south east asia OR **asia, western** OR western asia OR **europe, eastern** OR east europe OR eastern europe OR **developing country** OR developing countries OR developing nation? OR developing population? OR developing world OR less developed countr* OR less developed nation? OR less developed population? OR less developed world OR lesser developed countr* OR lesser developed nation? OR lesser developed population? OR lesser developed world OR under developed countr* OR under developed nation? OR under developed population? OR under developed world OR underdeveloped countr* OR underdeveloped nation? OR underdeveloped population? OR underdeveloped world OR middle income countr* OR middle income nation? OR middle income population? OR low income countr* OR low income nation? OR low income population? OR lower income countr* OR lower income nation? OR lower income population? OR underserved countr* OR underserved nation? OR underserved population? OR underserved world OR under served countr* OR under served nation? OR under served population? OR under served world OR deprived countr* OR deprived nation? OR deprived population? OR deprived world OR poor countr* OR poor nation? OR poor population? OR poor world OR poorer countr* OR poorer nation? OR poorer population? OR poorer world OR developing econom* OR less developed econom* OR lesser developed econom* OR under developed econom* OR underdeveloped econom* OR middle income econom* OR low income econom* OR lower income econom* OR low gdp OR low gnp OR low gross domestic OR low gross national OR lower gdp OR lower gnp OR lower gross domestic OR lower gross national OR lmic OR lmics OR third world OR lami countr* OR transitional countr* OR emerging economies OR emerging nation?).ti,ab,sh,kw.
29[a]	(afghan OR afghans OR afghani OR albanian? algerian? OR american samoan? OR angolan? OR antiguan? OR barbudan? OR argentine? OR argentinian? OR argentinean? OR armenian? OR aruban? OR azerbaijani? OR bahraini? OR bangladeshi? OR bangalees OR bajan? OR belarusian? OR byelorussian? OR belizean? OR beninese? OR bhutanese OR bolivian? OR bosnian? OR botswana OR batswana OR brazilian? OR brasilian? OR bulgarian? OR burkinabe OR burkinese OR burundian? OR cape verdean? OR cabo verdean? OR cambodian? OR khmer OR cameroonian? OR central african? OR chadian? OR chilean? OR chinese OR colombian? OR comorian? OR congolese OR costa rican? OR ivorian? OR croatian? OR cuban? OR cypriot? OR czech? OR djiboutian? OR dominican? OR ecuadorian? OR egyptian? OR salvadoran? OR equatorial guinean? OR equatoguinean? OR eritrean? OR estonian? OR swazi? OR swati? OR ethiopian? OR fijian OR gabonese OR gabonaise OR gambian? OR georgian? OR ghanaian? OR gibraltarian? OR greek? OR grenadian? OR guamanian? OR guatemalan? OR guinean? OR bissau guinean? OR guyanese OR haitian? OR honduran? OR hungarian? OR indian? OR indonesian? OR iranian? OR iraqian? OR iraqi? OR manx OR jamaican? OR jordanian? OR kazakhstani? OR kenyan? OR kirabati OR kirabatian? OR north korean? OR korean? OR kosovar? OR kosovan? OR kyrgyz* OR lao OR laotian? OR latvian? OR lebanese OR lesothan? OR lesothonian? OR mosotho OR basotho OR liberian? OR libyan? OR lithuanian? OR macanese OR macedonian? OR malagasy OR madagascan? OR malawian? OR malaysian? OR maldivian? OR malian? OR maltese OR marshallese? OR mauritanian? OR mauritian? OR mexican? OR micronesian? OR moldovan? OR mongolian? OR mongol OR montenegrin? OR moroccan? OR mozambican? OR burmese OR myanma OR namibian? OR nauruan? OR nepali OR nepalese OR netherlands antillean? OR nicaraguan? OR nigerien? OR nigerian? OR northern mariana islander? OR mariana? OR omani? OR pakistani? OR palauan? OR panamanian? OR papua new guinean? OR paraguayan? OR peruvian? OR philippine? OR philipine? OR phillipine? OR phillippine? OR filipino? OR filipina? OR polish OR pole OR poles OR portuguese OR puerto rican? OR romanian? OR russian? OR soviet people OR soviet population OR rwandan? OR rwandese OR ruandan? OR ruandese OR samoan? OR sao tomean? OR santomean? OR saudi arabian? OR saudi? OR senegalese OR serbian? OR montenegrin? OR seychellois OR seychelloise? OR sierra leonean? OR slovak? OR slovene? OR solomon islander? OR somali? OR south african? OR south sudanese OR sri lankan? OR ceylonese OR kittitian? OR nevisian? OR

	saint lucian? OR vincentian? OR sudanese OR surinamese? OR syrian? OR tajik? OR tajikistani? OR tanzanian? OR tanganyikan? OR thai OR timorese? OR togolese OR tongan? OR trinidadian? OR tobagonian? OR tunisian? OR turk? OR turkish OR turkmen? OR tuvaluan? OR ugandan? OR ukrainian? OR uruguayan? OR uzbek? OR vanuatu* OR venezuelan? OR vietnamese OR yemeni? OR yemenite? OR yemenese OR yugoslav? OR yugoslavian? OR zambian? OR zimbabwean?).ti,ab,sh,kw.
30	Mental health/
31	Mental disease
32	exp Drug dependence/
33	exp Anxiety disorder/
34	exp Mood disorder/
35	exp Suicidal behaviour/
36	Automutilation/
37	("mental health" or "mental illness*" or "mental disorder*" or "psychological disorder*" or "psychiatric disorder*" or psychopatholog* or anxiet* or depress* or "post-traumatic stress" or "posttraumatic stress" or PTSD or suicide or "self harm" or "substance use" or "substance abuse" or "alcohol use" or "alcohol abuse" or "drug use" or "drug abuse" or "mood disorder*").mp.
38	1 or 2 or 3 or 4 or 5 or 6 or 7 or 8 or 9 or 10 or 11 or 12 or 13 or 14 or 15 or 16 or 17 or 18 or 19 or 20 or 21 or 22 or 23 or 24 or 25 or 26 or 27
39	28 or 29
40	30 or 31 or 32 or 33 or 34 or 35 or 36 or 37
41	38 and 39 and 40
42	41 not ((exp animal/ or exp invertebrate/ or nonhuman/ or animal experiment/ or animal tissue/ or animal model/ or exp plant/ or exp fungus/) not exp human/)
43	Limit 42 to yr="2007 – 2019"
**TOTAL RESULTS: 4,159**	

[a]Used Cochrane's LMIC filters (https://epoc.cochrane.org/lmic-filters). Bolded terms represent subject headings.

Table S4: Detailed PsycInfo search strategy.

Line	Search Terms
1	Climate change/
2	Seasonal variations/
3	exp Temperature effects/
4	Natural disasters/
5	Atmospheric conditions/
6	Pollution/
7	("climate change*" or "climatic change*" or disaster* or "weather event*" or "extreme weather" or "global warming" or "climatic variability" or "air quality" or "water quality" or "pollution" or "land use" or "ecological change*" or "greenhouse gas*" or "extreme temperature*" or "cold wave*" or "heat wave*" or snow or snows or snowstorm* or ice or frost* or freez* or fog* or storm* or derecho* or hail* or thunderstorm* or lightning or rain* or tornado* or wind* or flood* or landslide* or avalanche* or rockfall* or mudflow* or "wave action*" or "rogue wave*" or seiche* or drought* or "glacial lake outburst*" or wildfire* or "forest fire*" or "land fire*" or "brush fire*" or "bush fire*" or "pasture fire*").mp.
8[a]	Developing countries/
9[a]	(afghanistan or albania or algeria or american samoa or angola or "antigua and barbuda" or antigua or barbuda or argentina or armenia or armenian or aruba or azerbaijan or bahrain or bangladesh or barbados or republic of belarus or belarus or byelarus or belorussia or byelorussian or belize or british honduras or benin or dahomey or bhutan or bolivia or "bosnia and herzegovina" or bosnia or herzegovina or botswana or bechuanaland or brazil or brasil or bulgaria or burkina faso or burkina fasso or upper volta or burundi or urundi or cabo verde or cape verde or cambodia or kampuchea or khmer republic or cameroon or cameron or cameroun or central african republic or ubangi shari or chad or chile or china or colombia or comoros or comoro islands or iles comores or mayotte or democratic republic of the congo or democratic republic congo or congo or zaire or costa rica or "cote d'ivoire" or "cote d' ivoire" or cote divoire or cote d ivoire or ivory coast or croatia or cuba or cyprus or czech republic or czechoslovakia or djibouti or french somaliland or dominica or dominican republic or ecuador or egypt or united arab republic or el salvador or equatorial guinea or spanish guinea or eritrea or estonia or eswatini or swaziland or ethiopia or fiji or gabon or gabonese republic or gambia or "georgia (republic)" or georgian or ghana or gold coast or gibraltar or greece or grenada or guam or guatemala or guinea or guinea bissau or guyana or british guiana or haiti or hispaniola or honduras or hungary or india or indonesia or timor or iran or iraq or isle of man or jamaica or jordan or kazakhstan or kazakh or kenya or "democratic people's republic of korea" or republic of korea or north korea or south korea or korea or kosovo or kyrgyzstan or kirghizia or kirgizstan or kyrgyz republic or kirghiz or laos or lao pdr or "lao people's democratic republic" or latvia or lebanon or lebanese republic or lesotho or basutoland or liberia or libya or libyan arab jamahiriya or lithuania or macau or macao or "macedonia (republic)" or macedonia or madagascar or malagasy republic or malawi or nyasaland or malaysia or malay federation or malaya federation or maldives or indian ocean islands or indian ocean or mali or malta or micronesia or federated states of micronesia or kiribati or marshall islands or nauru or northern mariana islands or palau or tuvalu or mauritania or mauritius or mexico or moldova or moldovian or mongolia or montenegro or "montenegro (republic)" or morocco or ifni or mozambique or portuguese east africa or myanmar or burma or namibia or nepal or netherlands antilles or nicaragua or niger or nigeria or oman or muscat or pakistan or panama or papua new guinea or new guinea or paraguay or peru or philippines or philipines or phillipines or phillippines or poland or "polish people's republic" or portugal or portuguese republic or puerto rico or romania or russia or russian federation or ussr or soviet union or union of soviet socialist republics or rwanda or ruanda or samoa or pacific islands or polynesia or samoan islands or navigator island or navigator islands or "sao tome and principe" or saudi arabia or senegal or serbia or seychelles or sierra leone or slovakia or slovak republic or slovenia or melanesia or solomon island or solomon islands or norfolk island or norfolk islands or somalia or south africa or south sudan or sri lanka or ceylon or "saint kitts and nevis" or "st. kitts and nevis" or saint lucia or "st. lucia" or "saint vincent and the grenadines" or saint vincent or "st. vincent" or grenadines or sudan or suriname or surinam or dutch guiana or netherlands guiana or syria or syrian arab republic or tajikistan or tadjikistan or tadzhikistan or tadzhik or tanzania or tanganyika or thailand or siam or timor leste or east timor or togo or togolese republic or tonga or "trinidad and tobago" or trinidad or tobago or tunisia or turkey or "turkey (republic)" or turkmenistan or turkmen or uganda or ukraine or uruguay or uzbekistan or uzbek or vanuatu or new hebrides or venezuela or vietnam or viet nam or middle east or west bank or gaza or palestine or yemen or yugoslavia or zambia or zimbabwe or northern rhodesia or global south or africa south of the sahara or "sub saharan africa" or subsaharan africa or africa, central or central africa or africa, northern or north africa or northern africa or magreb or maghrib or

	sahara or africa, southern or southern africa or africa, eastern or east africa or eastern africa or africa, western or west africa or western africa or west indies or indian ocean islands or caribbean region or caribbean islands or caribbean or central america or latin america or "south and central america" or south america or asia, central or central asia or asia, northern or north asia or northern asia or asia, southeastern or southeastern asia or south eastern asia or southeast asia or south east asia or asia, western or western asia or europe, eastern or east europe or eastern europe or developing country or developing countries or developing nation? or developing population? or developing world or less developed countr* or less developed nation? or less developed population? or less developed world or lesser developed countr* or lesser developed nation? or lesser developed population? or lesser developed world or under developed countr* or under developed nation? or under developed population? or under developed world or underdeveloped countr* or underdeveloped nation? or underdeveloped population? or underdeveloped world or middle income countr* or middle income nation? or middle income population? or low income countr* or low income nation? or low income population? or lower income countr* or lower income nation? or lower income population? or underserved countr* or underserved nation? or underserved population? or underserved world or under served countr* or under served nation? or under served population? or under served world or deprived countr* or deprived nation? or deprived population? or deprived world or poor countr* or poor nation? or poor population? or poor world or poorer countr* or poorer nation? or poorer population? or poorer world or developing econom* or less developed econom* or lesser developed econom* or under developed econom* or underdeveloped econom* or middle income econom* or low income econom* or lower income econom* or low gdp or low gnp or low gross domestic or low gross national or lower gdp or lower gnp or lower gross domestic or lower gross national or lmic or lmics or third world or lami countr* or transitional countr* or emerging economies or emerging nation?).mp.
10[a]	(afghan or afghans or afghani or albanian? algerian? or american samoan? or angolan? or antiguan? or barbudan? or argentine? or argentinian? or argentinean? or armenian? or aruban? or azerbaijani? or bahraini? or bangladeshi? or bangalees or bajan? or belarusian? or byelorussian? or belizean? or beninese? or bhutanese or bolivian? or bosnian? or botswana or batswana or brazilian? or brasilian? or bulgarian? or burkinabe or burkinese or burundian? or cape verdean? or cabo verdean? or cambodian? or khmer or cameroonian? or central african? or chadian? or chilean? or chinese or colombian? or comorian? or congolese or costa rican? or ivorian? or croatian? or cuban? or cypriot? or czech? or djiboutian? or dominican? or ecuadorian? or egyptian? or salvadoran? or equatorial guinean? or equatoguinean? or eritrean? or estonian? or swazi? or swati? or ethiopian? or fijian or gabonese or gabonaise or gambian? or georgian? or ghanaian? or gibraltarian? or greek? or grenadian? or guamanian? or guatemalan? or guinean? or bissau guinean? or guyanese or haitian? or honduran? or hungarian? or indian? or indonesian? or iranian? or iraqian? or iraqi? or manx or jamaican? or jordanian? or kazakhstani? or kenyan? or kirabati or kirabatian? or north korean? or korean? or kosovar? or kosovan? or kyrgyz* or lao or laotian? or latvian? or lebanese or lesothan? or lesothonian? or mosotho or basotho or liberian? or libyan? or lithuanian? or macanese or macedonian? or malagasy or madagascan? or malawian? or malaysian? or maldivian? or malian? or maltese or marshallese? or mauritanian? or mauritian? or mexican? or micronesian? or moldovan? or mongolian? or mongol or montenegrin? or moroccan? or mozambican? or burmese or myanma or namibian? or nauruan? or nepali or nepalese or netherlands antillean? or nicaraguan? or nigerien? or nigerian? or northern mariana islander? or mariana? or omani? or pakistani? or palauan? or panamanian? or papua new guinean? or paraguayan? or peruvian? or philippine? or philipine? or phillipine? or phillippine? or filipino? or filipina? or polish or pole or poles or portuguese or puerto rican? or romanian? or russian? or soviet people or soviet population or rwandan? or rwandese or ruandan? or ruandese or samoan? or sao tomean? or santomean? or saudi arabian? or saudi? or senegalese or serbian? or montenegrin? or seychellois or seychelloise? or sierra leonean? or slovak? or slovene? or solomon islander? or somali? or south african? or south sudanese or sri lankan? or ceylonese or kittitian? or nevisian? or saint lucian? or vincentian? or sudanese or surinamese? or syrian? or tajik? or tajikistani? or tanzanian? or tanganyikan? or thai or timorese? or togolese or tongan? or trinidadian? or tobagonian? or tunisian? or turk? or turkish or turkmen? or tuvaluan? or ugandan? or ukrainian? or uruguayan? or uzbek? or vanuatu* or venezuelan? or vietnamese or yemeni? or yemenite? or yemenese or yugoslav? or yugoslavian? or zambian? or zimbabwean?).mp.
11	Mental health/
12	Mental disorders/
13	exp Affective disorders/
14	exp Anxiety disorders/
15	Metal disorders due to general medical conditions/
16	exp Sleep wake disorders/
17	exp “Stress and trauma related disorders”/
18	exp “Substance use disorders”/
19	exp Suicide/
20	exp Self-injurious behaviour/
21	("mental health" or "mental illness*" or "mental disorder*" or "psychological disorder*" or "psychiatric disorder*" or psychopatholog* or anxiet* or depress* or "post-traumatic stress" or "posttraumatic stress" or PTSD or suicide or "self harm" or "substance use" or "substance abuse" or "alcohol use" or "alcohol abuse" or "drug use" or "drug abuse" or "mood disorder*").mp.
22	1 or 2 or 3 or 4 or 5 or 6 or 7
23	8 or 9 or 10
24	11 or 12 or 13 or 14 or 15 or 16 or 17 or 18 or 19 or 20 or 21
25	22 and 23 and 24
26	Limit 25 to human
27	25 not ((animals/ or animal models/) not 26)
28	Limit 27 to yr=”2007 – 2019”
**TOTAL RESULTS: 1,875**	

[a]Cochrane’s LMIC filter not available for PsycInfo. As such, the filter for MEDLINE was adapted for use here.

Table S5: Detailed Global Health search strategy.

Line	Search Terms
1	Climate change/
2	Climatic factors/
3	exp climate/

4	exp Climatic seasons/
5	Greenhouse effect/
6	Natural disasters/
7	Weather/
8	Avalanches/
9	exp Storms/
10	Drought/
11	Floods/
12	Wildfire/
13	exp Environmental temperature/
14	Frozen conditions/
15	exp Precipitation/
16	Cold/
17	Heat/
18	Lightning/
19	Wind/
20	Frost/
21	Ice/
22	Air pollution/
23	exp Air pollutants/
24	Air quality/
25	exp Water pollution/
26	Water quality/
27	Soil pollution/
28	("climate change*" or "climatic change*" or disaster* or "weather event*" or "extreme weather" or "global warming" or "climatic variability" or "air quality" or "water quality" or "pollution" or "land use" or "ecological change*" or "greenhouse gas*" or "extreme temperature*" or "cold wave*" or "heat wave*" or snow or snows or snowstorm* or ice or frost* or freez* or fog* or storm* or derecho* or hail* or thunderstorm* or lightning or rain* or tornado* or wind* or flood* or landslide* or avalanche* or rockfall* or mudflow* or "wave action*" or "rogue wave*" or seiche* or drought* or "glacial lake outburst*" or wildfire* or "forest fire*" or "land fire*" or "brush fire*" or "bush fire*" or "pasture fire*").mp.
29[a]	Developing countries/
30[a]	Least developed countries/
31[a]	exp Afghanistan/ or exp Burkina Faso/ or exp Burundi/ or exp Central African Republic/ or exp Chad/ or exp Congo Democratic Republic/ or exp Eritrea/ or exp Ethiopia/ or exp Gambia/ or exp Guinea/ or exp Guinea-Bissau/ or exp Haiti/ or exp North Korea/ or exp Liberia/ or exp Madagascar/ or exp Malawi/ or exp Mali/ or exp Mozambique/ or exp Niger/ or exp Rwanda/ or exp Sierra Leone/ or exp Somalia/ or exp Sudan/ or exp South Sudan/ or exp Syrian Arab Republic/ or exp Tajikistan/ or exp Togo/ or exp Uganda/ or exp Yemen/ or exp Angola/ or exp Algeria/ or exp Bangladesh/ or exp Benin/ or exp Bhutan/ or exp Bolivia/ or exp Cape Verde/ or exp Cambodia/ or exp Cameroon/ or exp Comoros/ or exp Congo/ or exp Cote d'Ivoire/ or exp Djibouti/ or exp Egypt/ or exp El Salvador/ or exp Ghana/ or exp Honduras/ or exp India/ or exp Kenya/ or exp Kiribati/ or exp Kyrgyzstan/ or exp Laos/ or exp Lesotho/ or exp Mauritania/ or exp "Federated States of Micronesia"/ or exp Moldova/ or exp Mongolia/ or exp Morocco/ or exp Myanmar/ or exp Nepal/ or exp Nicaragua/ or exp Nigeria/ or exp Pakistan/ or exp Papua New Guinea/ or exp Philippines/ or exp "Sao Tome and Principe"/ or exp Senegal/ or exp Solomon Islands/ or exp Sri Lanka/ or exp Tanzania/ or exp East Timor/ or exp Tunisia/ or exp Ukraine/ or exp Uzbekistan/ or exp Vanuatu/ or exp Viet Nam/ or exp Palestine/ or exp "Gaza (Palestine)"/ or exp Zambia/ or exp Zimbabwe/ or exp Albania/ or exp American Samoa/ or exp Argentina/ or exp Armenia/ or exp Azerbaijan/ or exp Belarus/ or exp Belize/ or exp "Bosnia and Herzegovina"/ or exp Botswana/ or exp Brazil/ or exp Bulgaria/ or exp China/ or exp Colombia/ or exp Costa Rica/ or exp Cuba/ or exp Dominica/ or exp Dominican Republic/ or exp Equatorial Guinea/ or exp Ecuador/ or exp Fiji/ or exp Gabon/ or exp "republic of georgia"/ or exp Grenada/ or exp Guatemala/ or exp Guyana/ or exp Indonesia/ or exp Iran/ or exp Iraq/ or exp Jamaica/ or exp Jordan/ or exp Kazakhstan/ or exp Kosovo/ or exp Lebanon/ or exp Libyan Arab Jamahiriya/ or exp Malaysia/ or exp Maldives/ or exp Marshall Islands/ or exp Mexico/ or exp Montenegro/ or exp Namibia/ or exp "Republic of North Macedonia"/ or exp Paraguay/ or exp Peru/ or exp Russian Federation/ or exp Samoa/ or exp Serbia/ or exp South Africa/ or exp Saint Lucia/ or exp "Saint Vincent and the Grenadines"/ or exp Suriname/ or exp Thailand/ or exp Tonga/ or exp Turkey/ or exp Turkmenistan/ or exp Tuvalu/ or exp Venezuela/ or exp Swaziland/
32[a]	(afghanistan or albania or algeria or american samoa or angola or "antigua and barbuda" or antigua or barbuda or argentina or armenia or armenian or aruba or azerbaijan or bahrain or bangladesh or barbados or republic of belarus or belarus or byelarus or belorussia or byelorussian or belize or british honduras or benin or dahomey or bhutan or bolivia or "bosnia and herzegovina" or bosnia or herzegovina or botswana or bechuanaland or brazil or brasil or bulgaria or burkina faso or burkina fasso or upper volta or burundi or urundi or cabo verde or cape verde or cambodia or kampuchea or khmer republic or cameroon or cameron or cameroun or central african republic or ubangi shari or chad or chile or china or colombia or comoros or comoro islands or iles comores or mayotte or democratic republic of the congo or democratic republic congo or congo or zaire or costa rica or "cote d’ivoire" or "cote d’ ivoire" or cote divoire or cote d ivoire or ivory coast or croatia or cuba or cyprus or czech republic or czechoslovakia or djibouti or french somaliland or dominica or dominican republic or ecuador or egypt or united arab republic or el salvador or equatorial guinea or spanish guinea or eritrea or estonia or eswatini or swaziland or ethiopia or fiji or gabon or gabonese republic or gambia or "georgia (republic)" or georgian or ghana or gold coast or gibraltar or greece or grenada or guam or guatemala or guinea or guinea bissau or guyana or british guiana or haiti or hispaniola or honduras or hungary or india or indonesia or timor or iran or iraq or isle of man or jamaica or jordan or kazakhstan or kazakh or kenya or "democratic people’s republic of korea" or republic of korea or north korea or south korea or korea or kosovo or kyrgyzstan or kirghizia or kirgizstan or kyrgyz republic or kirghiz or laos or lao pdr or "lao people's democratic republic" or latvia or lebanon or lebanese republic or lesotho or basutoland or liberia or libya or libyan arab jamahiriya or lithuania or macau or macao or "macedonia (republic)" or macedonia or madagascar or malagasy republic or malawi or nyasaland or malaysia or malay federation or malaya federation or maldives or indian ocean islands or indian ocean or mali or malta or micronesia or federated states of micronesia or kiribati or marshall islands or nauru or northern mariana islands or palau or tuvalu or mauritania or mauritius or mexico or moldova or moldovian or mongolia or montenegro or "montenegro (republic)" or morocco or ifni or mozambique or portuguese east africa or myanmar or burma or namibia or nepal or netherlands antilles or nicaragua or niger or nigeria or oman or muscat or pakistan or panama or papua new guinea or new guinea or paraguay or peru or philippines or philipines or phillipines or phillippines or poland or "polish people's republic" or portugal or portuguese republic or puerto rico or romania or russia or russian federation or ussr or soviet union or union of soviet socialist republics or rwanda or ruanda or samoa or pacific islands or polynesia or samoan islands or navigator island or navigator islands or

	"sao tome and principe" or saudi arabia or senegal or serbia or seychelles or sierra leone or slovakia or slovak republic or slovenia or melanesia or solomon island or solomon islands or norfolk island or norfolk islands or somalia or south africa or south sudan or sri lanka or ceylon or "saint kitts and nevis" or "st. kitts and nevis" or saint lucia or "st. lucia" or "saint vincent and the grenadines" or saint vincent or "st. vincent" or grenadines or sudan or suriname or surinam or dutch guiana or netherlands guiana or syria or syrian arab republic or tajikistan or tadjikistan or tadzhikistan or tadzhik or tanzania or tanganyika or thailand or siam or timor leste or east timor or togo or togolese republic or tonga or "trinidad and tobago" or trinidad or tobago or tunisia or turkey or "turkey (republic)" or turkmenistan or turkmen or uganda or ukraine or uruguay or uzbekistan or uzbek or vanuatu or new hebrides or venezuela or vietnam or viet nam or middle east or west bank or gaza or palestine or yemen or yugoslavia or zambia or zimbabwe or northern rhodesia or global south or africa south of the sahara or "sub saharan africa" or subsaharan africa or africa, central or central africa or africa, northern or north africa or northern africa or magreb or maghrib or sahara or africa, southern or southern africa or africa, eastern or east africa or eastern africa or africa, western or west africa or western africa or west indies or indian ocean islands or caribbean region or caribbean islands or caribbean or central america or latin america or "south and central america" or south america or asia, central or central asia or asia, northern or north asia or northern asia or asia, southeastern or southeastern asia or south eastern asia or southeast asia or south east asia or asia, western or western asia or europe, eastern or east europe or eastern europe or developing country or developing countries or developing nation? or developing population? or developing world or less developed countr* or less developed nation? or less developed population? or less developed world or lesser developed countr* or lesser developed nation? or lesser developed population? or lesser developed world or under developed countr* or under developed nation? or under developed population? or under developed world or underdeveloped countr* or underdeveloped nation? or underdeveloped population? or underdeveloped world or middle income countr* or middle income nation? or middle income population? or low income countr* or low income nation? or low income population? or lower income countr* or lower income nation? or lower income population? or underserved countr* or underserved nation? or underserved population? or underserved world or under served countr* or under served nation? or under served population? or under served world or deprived countr* or deprived nation? or deprived population? or deprived world or poor countr* or poor nation? or poor population? or poor world or poorer countr* or poorer nation? or poorer population? or poorer world or developing econom* or less developed econom* or lesser developed econom* or under developed econom* or underdeveloped econom* or middle income econom* or low income econom* or lower income econom* or low gdp or low gnp or low gross domestic or low gross national or lower gdp or lower gnp or lower gross domestic or lower gross national or lmic or lmics or third world or lami countr* or transitional countr* or emerging economies or emerging nation?).mp.
33[a]	(afghan or afghans or afghani or albanian? algerian? or american samoan? or angolan? or antiguan? or barbudan? or argentine? or argentinian? or argentinean? or armenian? or aruban? or azerbaijani? or bahraini? or bangladeshi? or bangalees or bajan? or belarusian? or byelorussian? or belizean? or beninese? or bhutanese or bolivian? or bosnian? or botswana or batswana or brazilian? or brasilian? or bulgarian? or burkinabe or burkinese or burundian? or cape verdean? or cabo verdean? or cambodian? or khmer or cameroonian? or central african? or chadian? or chilean? or chinese or colombian? or comorian? or congolese or costa rican? or ivorian? or croatian? or cuban? or cypriot? or czech? or djiboutian? or dominican? or ecuadorian? or egyptian? or salvadoran? or equatorial guinean? or equatoguinean? or eritrean? or estonian? or swazi? or swati? or ethiopian? or fijian or gabonese or gabonaise or gambian? or georgian? or ghanaian? or gibraltarian? or greek? or grenadian? or guamanian? or guatemalan? or guinean? or bissau guinean? or guyanese or haitian? or honduran? or hungarian? or indian? or indonesian? or iranian? or iraqian? or iraqi? or manx or jamaican? or jordanian? or kazakhstani? or kenyan? or kirabati or kirabatian? or north korean? or korean? or kosovar? or kosovan? or kyrgyz* or lao or laotian? or latvian? or lebanese or lesothan? or lesothonian? or mosotho or basotho or liberian? or libyan? or lithuanian? or macanese or macedonian? or malagasy or madagascan? or malawian? or malaysian? or maldivian? or malian? or maltese or marshallese? or mauritanian? or mauritian? or mexican? or micronesian? or moldovan? or mongolian? or mongol or montenegrin? or moroccan? or mozambican? or burmese or myanma or namibian? or nauruan? or nepali or nepalese or netherlands antillean? or nicaraguan? or nigerien? or nigerian? or northern mariana islander? or mariana? or omani? or pakistani? or palauan? or panamanian? or papua new guinean? or paraguayan? or peruvian? or philippine? or philipine? or phillipine? or phillippine? or filipino? or filipina? or polish or pole or poles or portuguese or puerto rican? or romanian? or russian? or soviet people or soviet population or rwandan? or rwandese or ruandan? or ruandese or samoan? or sao tomean? or santomean? or saudi arabian? or saudi? or senegalese or serbian? or montenegrin? or seychellois or seychelloise? or sierra leonean? or slovak? or slovene? or solomon islander? or somali? or south african? or south sudanese or sri lankan? or ceylonese or kittitian? or nevisian? or saint lucian? or vincentian? or sudanese or surinamese? or syrian? or tajik? or tajikistani? or tanzanian? or tanganyikan? or thai or timorese? or togolese or tongan? or trinidadian? or tobagonian? or tunisian? or turk? or turkish or turkmen? or tuvaluan? or ugandan? or ukrainian? or uruguayan? or uzbek? or vanuatu* or venezuelan? or vietnamese or yemeni? or yemenite? or yemenese or yugoslav? or yugoslavian? or zambian? or zimbabwean?).mp.
34	Mental health/
35	exp Mental disorders/
36	exp Substance abuse/
37	Suicide/
38	("mental health" or "mental illness*" or "mental disorder*" or "psychological disorder*" or "psychiatric disorder*" or psychopatholog* or anxiet* or depress* or "post-traumatic stress" or "posttraumatic stress" or PTSD or suicide or "self harm" or "substance use" or "substance abuse" or "alcohol use" or "alcohol abuse" or "drug use" or "drug abuse" or "mood disorder*").mp.
39	1 or 2 or 3 or 4 or 5 or 6 or 7 or 8 or 9 or 10 or 11 or 12 or 13 or 14 or 15 or 16 or 17 or 18 or 19 or 20 or 21 or 22 or 23 or 24 or 25 or 26 or 27 or 28
40	29 or 30 or 31 or 32 or 33
41	34 or 35 or 36 or 37 or 38
42	39 and 40 and 41
43	42 not ((exp Plants/ or exp Animals/ or exp Fungi/) not exp Man/)
44	Limit 43 to yr="2007 – 2019"
**TOTAL RESULTS: 1,245**	

[a]Cochrane's LMIC filter not available for Global Health. As such, the filter for MEDLINE was adapted for use here.

Table S6: Detailed Sociological Abstracts search strategy.

Line	Search Terms
1	(MAINSUBJECT.EXACT("Climate Change")

2	MAINSUBJECT.EXACT("Weather")
3	MAINSUBJECT.EXACT("Environmental Degradation")
4	MAINSUBJECT.EXACT("Natural disasters")
5	MAINSUBJECT.EXACT("Seasonal variations")
6	MAINSUBJECT.EXACT.EXPLODE("Pollution")
7	noft("climate change*" OR "climatic change*" OR disaster* OR "weather event*" OR "extreme weather" OR "global warming" OR "climatic variability" OR "air quality" OR "water quality" OR "pollution" OR "land use" OR "ecological change*" OR "greenhouse gas*" OR "extreme temperature*" OR "cold wave*" OR "heat wave*" OR snow OR snows OR snowstorm* OR ice OR frost* OR freez* OR fog* OR storm* OR derecho* OR hail* OR thunderstorm* OR lightning OR rain* OR tornado* OR wind* OR flood* OR landslide* OR avalanche* OR rockfall* OR mudflow* OR "wave action*" OR "rogue wave*" OR seiche* OR drought* OR "glacial lake outburst*" OR wildfire* OR "forest fire*" OR "land fire*" OR "brush fire*" OR "bush fire*" OR "pasture fire*"))
8[a]	(MAINSUBJECT.EXACT("Low Income Areas")
9[a]	MAINSUBJECT.EXACT("Developing countries")
10[a]	noft(afghanistan OR albania OR algeria OR american samoa OR angola OR "antigua and barbuda" OR antigua OR barbuda OR argentina OR armenia OR armenian OR aruba OR azerbaijan OR bahrain OR bangladesh OR barbados OR republic of belarus OR belarus OR byelarus OR belorussia OR byelorussian OR belize OR british honduras OR benin OR dahomey OR bhutan OR bolivia OR "bosnia and herzegovina" OR bosnia OR herzegovina OR botswana OR bechuanaland OR brazil OR brasil OR bulgaria OR burkina faso OR burkina fasso OR upper volta OR burundi OR urundi OR cabo verde OR cape verde OR cambodia OR kampuchea OR khmer republic OR cameroon OR cameron OR cameroun OR central african republic OR ubangi shari OR chad OR chile OR china OR colombia OR comoros OR comoro islands OR iles comores OR mayotte OR democratic republic of the congo OR democratic republic congo OR congo OR zaire OR costa rica OR "cote d’ivoire" OR "cote d’ ivoire" OR cote divoire OR cote d ivoire OR ivory coast OR croatia OR cuba OR cyprus OR czech republic OR czechoslovakia OR djibouti OR french somaliland OR dominica OR dominican republic OR ecuador OR egypt OR united arab republic OR el salvador OR equatorial guinea OR spanish guinea OR eritrea OR estonia OR eswatini OR swaziland OR ethiopia OR fiji OR gabon OR gabonese republic OR gambia OR "georgia (republic)" OR georgian OR ghana OR gold coast OR gibraltar OR greece OR grenada OR guam OR guatemala OR guinea OR guinea bissau OR guyana OR british guiana OR haiti OR hispaniola OR honduras OR hungary OR india OR indonesia OR timor OR iran OR iraq OR isle of man OR jamaica OR jordan OR kazakhstan OR kazakh OR kenya OR "democratic people’s republic of korea" OR republic of korea OR north korea OR south korea OR korea OR kosovo OR kyrgyzstan OR kirghizia OR kirgizstan OR kyrgyz republic OR kirghiz OR laos OR lao pdr OR "lao people's democratic republic" OR latvia OR lebanon OR lebanese republic OR lesotho OR basutoland OR liberia OR libya OR libyan arab jamahiriya OR lithuania OR macau OR macao OR "macedonia (republic)" OR macedonia OR madagascar OR malagasy republic OR malawi OR nyasaland OR malaysia OR malay federation OR malaya federation OR maldives OR indian ocean islands OR indian ocean OR mali OR malta OR micronesia OR federated states of micronesia OR kiribati OR marshall islands OR nauru OR northern mariana islands OR palau OR tuvalu OR mauritania OR mauritius OR mexico OR moldova OR moldovian OR mongolia OR montenegro OR "montenegro (republic)" OR morocco OR ifni OR mozambique OR portuguese east africa OR myanmar OR burma OR namibia OR nepal OR netherlands antilles OR nicaragua OR niger OR nigeria OR oman OR muscat OR pakistan OR panama OR papua new guinea OR new guinea OR paraguay OR peru OR philippines OR philipines OR phillipines OR phillippines OR poland OR "polish people's republic" OR portugal OR portuguese republic OR puerto rico OR romania OR russia OR russian federation OR ussr OR soviet union OR union of soviet socialist republics OR rwanda OR ruanda OR samoa OR pacific islands OR polynesia OR samoan islands OR navigator island OR navigator islands OR "sao tome and principe" OR saudi arabia OR senegal OR serbia OR seychelles OR sierra leone OR slovakia OR slovak republic OR slovenia OR melanesia OR solomon island OR solomon islands OR norfolk island OR norfolk islands OR somalia OR south africa OR south sudan OR sri lanka OR ceylon OR "saint kitts and nevis" OR "st. kitts and nevis" OR saint lucia OR "st. lucia" OR "saint vincent and the grenadines" OR saint vincent OR "st. vincent" OR grenadines OR sudan OR suriname OR surinam OR dutch guiana OR netherlands guiana OR syria OR syrian arab republic OR tajikistan OR tadjikistan OR tadzhikistan OR tadzhik OR tanzania OR tanganyika OR thailand OR siam OR timor leste OR east timor OR togo OR togolese republic OR tonga OR "trinidad and tobago" OR trinidad OR tobago OR tunisia OR turkey OR "turkey (republic)" OR turkmenistan OR turkmen OR uganda OR ukraine OR uruguay OR uzbekistan OR uzbek OR vanuatu OR new hebrides OR venezuela OR vietnam OR viet nam OR middle east OR west bank OR gaza OR palestine OR yemen OR yugoslavia OR zambia OR zimbabwe OR northern rhodesia OR global south OR africa south of the sahara OR "sub saharan africa" OR subsaharan africa OR africa, central OR central africa OR africa, northern OR north africa OR northern africa OR magreb OR maghrib OR sahara OR africa, southern OR southern africa OR africa, eastern OR east africa OR eastern africa OR africa, western OR west africa OR western africa OR west indies OR indian ocean islands OR caribbean region OR caribbean islands OR caribbean OR central america OR latin america OR "south and central america" OR south america OR asia, central OR central asia OR asia, northern OR north asia OR northern asia OR asia, southeastern OR southeastern asia OR south eastern asia OR southeast asia OR south east asia OR asia, western OR western asia OR europe, eastern OR east europe OR eastern europe OR developing country OR developing countries OR developing nation? OR developing population? OR developing world OR less developed countr* OR less developed nation? OR less developed population? OR less developed world OR lesser developed countr* OR lesser developed nation? OR lesser developed population? OR lesser developed world OR under developed countr* OR under developed nation? OR under developed population? OR under developed world OR underdeveloped countr* OR underdeveloped nation? OR underdeveloped population? OR underdeveloped world OR middle income countr* OR middle income nation? OR middle income population? OR low income countr* OR low income nation? OR low income population? OR lower income countr* OR lower income nation? OR lower income population? OR underserved countr* OR underserved nation? OR underserved population? OR underserved world OR under served countr* OR under served nation? OR under served population? OR under served world OR deprived countr* OR deprived nation? OR deprived population? OR deprived world OR poor countr* OR poor nation? OR poor population? OR poor world OR poorer countr* OR poorer nation? OR poorer population? OR poorer world OR developing econom* OR less developed econom* OR lesser developed econom* OR under developed econom* OR underdeveloped econom* OR middle income econom* OR low income econom* OR lower income econom* OR low gdp OR low gnp OR low gross domestic OR low gross national OR lower gdp OR lower gnp OR lower gross domestic OR lower gross national OR lmic OR lmics OR third world OR lami countr* OR transitional countr* OR emerging economies OR emerging nation?)
11[a]	noft(afghan OR afghans OR afghani OR albanian? algerian? OR american samoan? OR angolan? OR antiguan? OR barbudan? OR argentine? OR argentinian? OR argentinean? OR armenian? OR aruban? OR azerbaijani? OR bahraini? OR bangladeshi? OR bangalees OR bajan? OR belarusian? OR byelorussian? OR belizean? OR beninese? OR bhutanese OR bolivian? OR bosnian? OR botswana OR batswana OR brazilian? OR brasilian? OR bulgarian? OR burkinabe OR burkinese OR burundian? OR cape verdean? OR cabo verdean? OR cambodian? OR khmer OR cameroonian? OR central african? OR chadian? OR chilean? OR chinese OR colombian? OR comorian? OR congolese OR costa rican? OR ivorian? OR croatian? OR cuban? OR cypriot? OR czech? OR djiboutian? OR dominican? OR ecuadorian? OR egyptian? OR salvadoran? OR equatorial guinean? OR equatoguinean? OR eritrean? OR estonian? OR swazi? OR swati? OR ethiopian? OR fijian OR gabonese OR gabonaise OR gambian? OR georgian? OR ghanaian? OR gibraltarian? OR greek? OR grenadian? OR guamanian? OR guatemalan? OR guinean? OR bissau guinean? OR guyanese OR haitian? OR honduran? OR hungarian? OR indian? OR indonesian? OR iranian? OR iraqian? OR iraqi? OR

	manx OR jamaican? OR jordanian? OR kazakhstani? OR kenyan? OR kirabati OR kirabatian? OR north korean? OR korean? OR kosovar? OR kosovan? OR kyrgyz* OR lao OR laotian? OR latvian? OR lebanese OR lesothan? OR lesothonian? OR mosotho OR basotho OR liberian? OR libyan? OR lithuanian? OR macanese OR macedonian? OR malagasy OR madagascan? OR malawian? OR malaysian? OR maldivian? OR malian? OR maltese OR marshallese? OR mauritanian? OR mauritian? OR mexican? OR micronesian? OR moldovan? OR mongolian? OR mongol OR montenegrin? OR moroccan? OR mozambican? OR burmese OR myanma OR namibian? OR nauruan? OR nepali OR nepalese OR netherlands antillean? OR nicaraguan? OR nigerien? OR nigerian? OR northern mariana islander? OR mariana? OR omani? OR pakistani? OR palauan? OR panamanian? OR papua new guinean? OR paraguayan? OR peruvian? OR philippine? OR philipine? OR phillipine? OR phillippine? OR filipino? OR filipina? OR polish OR pole OR poles OR portuguese OR puerto rican? OR romanian? OR russian? OR soviet people OR soviet population OR rwandan? OR rwandese OR ruandan? OR ruandese OR samoan? OR sao tomean? OR santomean? OR saudi arabian? OR saudi? OR senegalese OR serbian? OR montenegrin? OR seychellois OR seychelloise? OR sierra leonean? OR slovak? OR slovene? OR solomon islander? OR somali? OR south african? OR south sudanese OR sri lankan? OR ceylonese OR kittitian? OR nevisian? OR saint lucian? OR vincentian? OR sudanese OR surinamese? OR syrian? OR tajik? OR tajikistani? OR tanzanian? OR tanganyikan? OR thai OR timorese? OR togolese OR tongan? OR trinidadian? OR tobagonian? OR tunisian? OR turk? OR turkish OR turkmen? OR tuvaluan? OR ugandan? OR ukrainian? OR uruguayan? OR uzbek? OR vanuatu* OR venezuelan? OR vietnamese OR yemeni? OR yemenite? OR yemenese OR yugoslav? OR yugoslavian? OR zambian? OR zimbabwean?))
12	MAINSUBJECT.EXACT("Mental Health")
13	MAINSUBJECT.EXACT("Mental illness")
14	MAINSUBJECT.EXACT("Anxiety")
15	MAINSUBJECT.EXACT.EXPLODE("Affective Illness")
16	MAINSUBJECT.EXACT("Posttraumatic Stress Disorder")
17	MAINSUBJECT.EXACT("Psychopathology")
18	MAINSUBJECT.EXACT.EXPLODE("Substance Abuse")
19	MAINSUBJECT.EXACT.EXPLODE("Self Destructive Behavior")
20	noft("mental health" OR "mental illness*" OR "mental disorder*" OR "psychological disorder*" OR "psychiatric disorder*" OR psychopatholog* OR anxiet* OR depress* OR "post-traumatic stress" OR "posttraumatic stress" OR PTSD OR suicide OR "self harm" OR "substance use" OR "substance abuse" OR "alcohol use" OR "alcohol abuse" OR "drug use" OR "drug abuse" OR "mood disorder*"))
21	1 or 2 or 3 or 4 or 5 or 6 or 7
22	8 or 9 or 10 or 11
23	12 or 13 or 14 or 15 or 16 or 17 or 18 or 19 or 20
24	21 and 22 and 23
25	24 pd(20070101-20191231)
**TOTAL RESULTS: 374**	

[a]Cochrane's LMIC filter not available for Sociological Abstracts. As such, the filter for MEDLINE was adapted for use here.

Table S7: Grey literature search strategy for targeted website browsing/searching.

Date	Organization Name and URL	Search Strategy?	# Search Results	# Refs Identified
25-Aug-20	American Psychological Association (https://www.apa.org/)	Yes	148	0
25-Aug-20	Centre for Disaster Preparedness and Management (http://www.uop.edu.pk/departments/?q=Centre-for-Disaster-Preparedness-and-Management)	No	N/A	0
25-Aug-20	Center for Global Development (https://www.cgdev.org/)	Yes	6,936	0
25-Aug-20	Centre for Research on the Epidemiology of Disasters (https://www.cred.be/)	No	N/A	0
25-Aug-20	Centre for Resource Management and Environmental Studies (https://www.cavehill.uwi.edu/cermes/home.aspx)	No	N/A	0
25-Aug-20	Copenhagen Center for Disaster Research (https://cope.ku.dk/)	No	N/A	0
25-Aug-20	Institute for Health Metrics and Evaluation (http://www.healthdata.org/)	Yes	Not reported	0
25-Aug-20	Inter-American Development Bank (https://www.iadb.org/en)	Yes	0	0
25-Aug-20	Intergovernmental Panel on Climate Change (https://www.ipcc.ch/)	Yes	0	0
25-Aug-20	Internal Displacement Monitoring Centre (https://www.internal-displacement.org/)	Yes	327	0
25-Aug-20	International Institute for Sustainable Development (https://www.iisd.org/)	Yes	0	0
25-Aug-20	International Monetary Fund (https://www.elibrary.imf.org/)	Yes	98[a]	0
25-Aug-20	International Research Institute for Climate and Society (https://iri.columbia.edu/)	Yes	0	0
13-Sep-20	Natural Hazards Center (https://hazards.colorado.edu/)	No	N/A	0
13-Sep-20	Oxfam International (https://www.oxfam.org/en)	Yes	0	0
13- Sep-20	Pan-American Health Organization (https://www.paho.org/en)	Yes	~1,480	0
13-Sep-20	Partners Enhancing Resilience for People Exposed to Risks (PERIPERI U) (https://www.riskreductionafrica.org/) – searched the Online Research Centre (http://lib.riskreductionafrica.org/)	Yes	937	0
13-Sep-20	Stockholm Environment Institute (https://www.sei.org/)	Yes	3,485[a]	0
13-Sep-20	The Global Climate and Health Alliance (http://climateandhealthalliance.org/)	Yes	0	0
13-Sep-20	United Nations (https://www.un.org/en/) – searched the Official Document System (https://documents.un.org/prod/ods.nsf/home.xsp)	Yes	0	0
13-Sep-20	United Nations Framework Convention on Climate Change (https://unfccc.int/)	Yes	337	0
13-Sep-20	United Nations High Commissioner for Refugees (https://www.unhcr.org/)	Yes	0	0
13-Sep-20	United Nations Office for Disaster Risk Reduction (https://www.undrr.org/)	Yes	501	0
13-Sep-20	University College London Hazard Centre (https://www.ucl.ac.uk/hazard-centre/)	No	N/A	0
13-Sep-20	Urban Climate Change Research Network (https://uccrn.ei.columbia.edu/)	Yes	1	0
13-Sep-20	World Bank Group (https://www.worldbank.org/) – searched the Open Knowledge Repository (https://openknowledge.worldbank.org/)	Yes	1,059	0
13-Sep-20	World Health Organization (https://www.who.int/) - searched WHOLIS (https://kohahq.searo.who.int/)	Yes	1	0
13-Sep-20	World Resources Institute (https://www.wri.org/)	Yes	1	0

[a]Used available filter for date of publication (2007-2019).

Notes: If the website contained a search feature, the following search strategy was used: (climate change OR natural disaster OR weather OR pollution) AND (anxiety OR depression OR stress OR suicide OR substance abuse). No country-specific terms were included in the search due to length.

Table S8: Grey literature search strategy for database searching.

Date	Database Name and URL	Search Strategy	# Search Results	# Refs Identified
**Grey Literature Databases**				
14-Sep-20	Google Scholar (https://scholar.google.ca/)	1. (climate change OR natural disaster OR weather OR pollution) AND (anxiety OR depression OR stress OR suicide OR substance abuse)<br>2. Custom date range 2007-2019	~51,800	0
14-Sep-20	OAIster (https://www.oclc.org/en/oaister.html)	1. kw:(climate change OR natural disaster OR weather OR pollution) AND (anxiety OR depression OR stress OR suicide OR substance abuse)<br>2. Custom date range 2007-2019	47	0
14-Sep-20	OpenGrey (http://www.opengrey.eu/)	1. (climate change OR natural disaster OR weather OR pollution) AND (anxiety OR depression OR stress OR suicide OR substance abuse)<br>2. Limit to English language[a]	139	0
14-Sep-20	The Grey Literature Report (https://www.greylit.org/)	1. (climate change OR natural disaster OR weather OR pollution) AND (anxiety OR depression OR stress OR suicide OR substance abuse)	0	0
14-Sep-20	ELDIS (specific to global development) (https://www.eldis.org/)	1. climate change AND mental health<br>2. Filter Documents only	14	0
**Conference Proceedings**				
14-Sep-20	BIOSIS Previews (https://www.ebsco.com/products/research-databases/biosis-previews)<br><br>-Used Web of Science's advanced search functions	1. TS=("climate change*" OR "climatic change*" OR disaster* OR "weather event*" OR "extreme weather" OR "global warming" OR "climatic variability" OR "air quality" OR "water quality" OR "pollution" OR "land use" OR "ecological change*" OR "greenhouse gas*" OR "extreme temperature*" OR "cold wave*" OR "heat wave*" OR snow OR snows OR snowstorm* OR ice OR frost* OR freez* OR fog* OR storm* OR derecho* OR hail* OR thunderstorm* OR lightning OR rain* OR tornado* OR wind* OR flood* OR landslide* OR avalanche* OR rockfall* OR mudflow* OR "wave action*" OR "rogue wave*" OR seiche* OR drought* OR "glacial lake outburst*" OR wildfire* OR "forest fire*" OR "land fire*" OR "brush fire*" OR "bush fire*" OR "pasture fire*"))<br>2. TS=(afghanistan OR albania OR algeria OR american samoa OR angola OR "antigua and barbuda" OR antigua OR barbuda OR argentina OR armenia OR armenian OR aruba OR azerbaijan OR bahrain OR bangladesh OR barbados OR republic of belarus OR belarus OR byelarus OR belorussia OR byelorussian OR belize OR british honduras OR benin OR dahomey OR bhutan OR bolivia OR "bosnia and herzegovina" OR bosnia OR herzegovina OR botswana OR bechuanaland OR brazil OR brasil OR bulgaria OR burkina faso OR burkina fasso OR upper volta OR burundi OR urundi OR cabo verde OR cape verde OR cambodia OR kampuchea OR khmer republic OR cameroon OR cameron OR cameroun OR central african republic OR ubangi shari OR chad OR chile OR china OR colombia OR comoros OR comoro islands OR iles comores OR mayotte OR democratic republic of the congo OR democratic republic congo OR congo OR zaire OR costa rica OR "cote d'ivoire" OR "cote d' ivoire" OR cote divoire OR cote d ivoire OR ivory coast OR croatia OR cuba OR cyprus OR czech republic OR czechoslovakia OR djibouti OR french somaliland OR dominica OR dominican republic OR ecuador OR egypt OR united arab republic OR el salvador OR equatorial guinea OR spanish guinea OR eritrea OR estonia OR eswatini OR swaziland OR ethiopia OR fiji OR gabon OR gabonese republic OR gambia OR "georgia (republic)" OR georgian OR ghana OR gold coast OR gibraltar OR greece OR grenada OR guam OR guatemala OR guinea OR guinea bissau OR guyana OR british guiana OR haiti OR hispaniola OR honduras OR hungary OR india OR indonesia OR timor OR iran OR iraq OR isle of man OR jamaica OR jordan OR kazakhstan OR kazakh OR kenya OR "democratic people's republic of korea" OR republic of korea OR north korea OR south korea OR korea OR kosovo OR kyrgyzstan OR kirghizia OR kirgizstan OR kyrgyz republic OR kirghiz OR laos OR lao pdr OR "lao people's democratic republic" OR latvia OR lebanon OR lebanese republic OR lesotho OR basutoland OR liberia OR libya OR libyan arab jamahiriya OR lithuania OR macau OR macao OR "macedonia (republic)" OR macedonia OR madagascar OR malagasy republic OR malawi OR nyasaland OR malaysia OR malay federation OR malaya federation OR maldives OR indian ocean islands OR indian ocean OR mali OR malta OR micronesia OR federated states of micronesia OR kiribati OR marshall islands OR nauru OR northern mariana islands OR palau OR tuvalu OR mauritania OR mauritius OR mexico OR moldova OR moldovian OR mongolia OR montenegro OR "montenegro (republic)" OR morocco OR ifni OR mozambique OR portuguese east africa OR myanmar OR burma OR namibia OR nepal OR netherlands antilles OR nicaragua OR niger OR nigeria OR oman OR muscat OR pakistan OR panama OR papua new guinea OR new guinea OR paraguay OR peru OR philippines OR philipines OR phillipines OR phillippines OR poland OR "polish people's republic" OR portugal OR portuguese republic OR puerto rico OR romania OR russia OR russian federation OR ussr OR soviet union OR union of soviet socialist republics OR rwanda OR ruanda OR samoa OR pacific islands OR polynesia OR samoan islands OR	98	0

		navigator island OR navigator islands OR "sao tome and principe" OR saudi arabia OR senegal OR serbia OR seychelles OR sierra leone OR slovakia OR slovak republic OR slovenia OR melanesia OR solomon island OR solomon islands OR norfolk island OR norfolk islands OR somalia OR south africa OR south sudan OR sri lanka OR ceylon OR "saint kitts and nevis" OR "st. kitts and nevis" OR saint lucia OR "st. lucia" OR "saint vincent and the grenadines" OR saint vincent OR "st. vincent" OR grenadines OR sudan OR suriname OR surinam OR dutch guiana OR netherlands guiana OR syria OR syrian arab republic OR tajikistan OR tadjikistan OR tadzhikistan OR tadzhik OR tanzania OR tanganyika OR thailand OR siam OR timor leste OR east timor OR togo OR togolese republic OR tonga OR "trinidad and tobago" OR trinidad OR tobago OR tunisia OR turkey OR "turkey (republic)" OR turkmenistan OR turkmen OR uganda OR ukraine OR uruguay OR uzbekistan OR uzbek OR vanuatu OR new hebrides OR venezuela OR vietnam OR viet nam OR middle east OR west bank OR gaza OR palestine OR yemen OR yugoslavia OR zambia OR zimbabwe OR northern rhodesia OR global south OR africa south of the sahara OR "sub saharan africa" OR subsaharan africa OR africa, central OR central africa OR africa, northern OR north africa OR northern africa OR magreb OR maghrib OR sahara OR africa, southern OR southern africa OR africa, eastern OR east africa OR eastern africa OR africa, western OR west africa OR western africa OR west indies OR indian ocean islands OR caribbean region OR caribbean islands OR caribbean OR central america OR latin america OR "south and central america" OR south america OR asia, central OR central asia OR asia, northern OR north asia OR northern asia OR asia, southeastern OR southeastern asia OR south eastern asia OR southeast asia OR south east asia OR asia, western OR western asia OR europe, eastern OR east europe OR eastern europe OR developing country OR developing countries OR developing nation? OR developing population? OR developing world OR less developed countr* OR less developed nation? OR less developed population? OR less developed world OR lesser developed countr* OR lesser developed nation? OR lesser developed population? OR lesser developed world OR under developed countr* OR under developed nation? OR under developed population? OR under developed world OR underdeveloped countr* OR underdeveloped nation? OR underdeveloped population? OR underdeveloped world OR middle income countr* OR middle income nation? OR middle income population? OR low income countr* OR low income nation? OR low income population? OR lower income countr* OR lower income nation? OR lower income population? OR underserved countr* OR underserved nation? OR underserved population? OR underserved world OR under served countr* OR under served nation? OR under served population? OR under served world OR deprived countr* OR deprived nation? OR deprived population? OR deprived world OR poor countr* OR poor nation? OR poor population? OR poor world OR poorer countr* OR poorer nation? OR poorer population? OR poorer world OR developing econom* OR less developed econom* OR lesser developed econom* OR under developed econom* OR underdeveloped econom* OR middle income econom* OR low income econom* OR lower income econom* OR low gdp OR low gnp OR low gross domestic OR low gross national OR lower gdp OR lower gnp OR lower gross domestic OR lower gross national OR lmic OR lmics OR third world OR lami countr* OR transitional countr* OR emerging economies OR emerging nation?)<br>3. TS=(afghan OR afghans OR afghani OR albanian? algerian? OR american samoan? OR angolan? OR antiguan? OR barbudan? OR argentine? OR argentinian? OR argentinean? OR armenian? OR aruban? OR azerbaijani? OR bahraini? OR bangladeshi? OR bangalees OR bajan? OR belarusian? OR byelorussian? OR belizean? OR beninese? OR bhutanese OR bolivian? OR bosnian? OR botswana OR batswana OR brazilian? OR brasilian? OR bulgarian? OR burkinabe OR burkinese OR burundian? OR cape verdean? OR cabo verdean? OR cambodian? OR khmer OR cameroonian? OR central african? OR chadian? OR chilean? OR chinese OR colombian? OR comorian? OR congolese OR costa rican? OR ivorian? OR croatian? OR cuban? OR cypriot? OR czech? OR djiboutian? OR dominican? OR ecuadorian? OR egyptian? OR salvadoran? OR equatorial guinean? OR equatoguinean? OR eritrean? OR estonian? OR swazi? OR swati? OR ethiopian? OR fijian OR gabonese OR gabonaise OR gambian? OR georgian? OR ghanaian? OR gibraltarian? OR greek? OR grenadian? OR guamanian? OR guatemalan? OR guinean? OR bissau guinean? OR guyanese OR haitian? OR honduran? OR hungarian? OR indian? OR indonesian? OR iranian? OR iraqian? OR iraqi? OR manx OR jamaican? OR jordanian? OR kazakhstani? OR kenyan? OR kirabati OR kirabatian? OR north korean? OR korean? OR kosovar? OR kosovan? OR kyrgyz* OR lao OR laotian? OR latvian? OR lebanese OR lesothan? OR lesothonian? OR mosotho OR basotho OR liberian? OR libyan? OR lithuanian? OR macanese OR macedonian? OR malagasy OR madagascan? OR malawian? OR malaysian? OR maldivian? OR malian? OR maltese OR marshallese? OR mauritanian? OR mauritian? OR mexican? OR micronesian? OR moldovan? OR mongolian? OR mongol OR montenegrin? OR moroccan? OR mozambican? OR burmese OR myanma OR namibian? OR nauruan? OR nepali OR nepalese OR netherlands antillean? OR nicaraguan? OR nigerien? OR nigerian? OR northern mariana islander? OR mariana? OR omani? OR pakistani? OR palauan? OR panamanian? OR papua new guinean? OR paraguayan? OR peruvian? OR philippine? OR philipine? OR phillipine? OR phillippine? OR filipino? OR filipina? OR polish OR pole OR poles OR portuguese OR puerto rican? OR romanian? OR russian? OR soviet people OR soviet population OR rwandan? OR rwandese OR ruandan? OR ruandese OR samoan? OR sao tomean? OR santomean? OR saudi arabian? OR saudi? OR senegalese OR serbian? OR montenegrin? OR seychellois OR seychelloise? OR sierra leonean? OR slovak? OR slovene? OR solomon islander? OR somali? OR south african? OR south sudanese OR sri lankan? OR ceylonese OR kittitian? OR nevisian? OR saint lucian? OR vincentian? OR sudanese OR surinamese? OR syrian? OR tajik? OR tajikistani? OR tanzanian? OR tanganyikan? OR thai OR timorese? OR togolese OR tongan? OR trinidadian? OR tobagonian? OR tunisian? OR turk? OR turkish OR		

		turkmen? OR tuvaluan? OR ugandan? OR ukrainian? OR uruguayan? OR uzbek? OR vanuatu* OR venezuelan? OR vietnamese OR yemeni? OR yemenite? OR yemenese OR yugoslav? OR yugoslavian? OR zambian? OR zimbabwean?))<br>4. TS=("mental health" OR "mental illness*" OR "mental disorder*" OR "psychological disorder*" OR "psychiatric disorder*" OR psychopatholog* OR anxiet* OR depress* OR "post-traumatic stress" OR "posttraumatic stress" OR PTSD OR suicide OR "self harm" OR "substance use" OR "substance abuse" OR "alcohol use" OR "alcohol abuse" OR "drug use" OR "drug abuse" OR "mood disorder*"))<br>5. #2 OR #3<br>6. #1 AND #4 AND #5<br>7. #6 Timespan=2007-2019<br>8. #7 AND LITERATURE TYPE: (Abstracts Only OR Meeting Abstract OR Meeting Address OR Meeting Paper OR Meeting Poster OR Meeting Report OR Meeting Slide OR Meeting Summary)		
**Dissertations and Theses**				
13-Sep-20	Center for Research Libraries (https://www.crl.edu/)	1. (climate change OR natural disaster OR weather OR pollution) AND (anxiety OR depression OR stress OR suicide OR substance abuse)	0	0
13-Sep-20	Networked Digital Library of Theses and Dissertations (http://search.ndltd.org/)	1. (climate change OR natural disaster OR weather OR pollution) AND (anxiety OR depression OR stress OR suicide OR substance abuse)<br>2. Limit publication year to 2007-2019<br>3. Limit to English language[a]	11,594	0
13-Sep-20	ProQuest Dissertations & Theses Global (https://about.proquest.com/products-services/pqdtglobal.html)<br><br>-Used ProQuest's advanced search functions	1. noft("climate change*" OR "climatic change*" OR disaster* OR "weather event*" OR "extreme weather" OR "global warming" OR "climatic variability" OR "air quality" OR "water quality" OR "pollution" OR "land use" OR "ecological change*" OR "greenhouse gas*" OR "extreme temperature*" OR "cold wave*" OR "heat wave*" OR snow OR snows OR snowstorm* OR ice OR frost* OR freez* OR fog* OR storm* OR derecho* OR hail* OR thunderstorm* OR lightning OR rain* OR tornado* OR wind* OR flood* OR landslide* OR avalanche* OR rockfall* OR mudflow* OR "wave action*" OR "rogue wave*" OR seiche* OR drought* OR "glacial lake outburst*" OR wildfire* OR "forest fire*" OR "land fire*" OR "brush fire*" OR "bush fire*" OR "pasture fire*"))<br>2. noft(afghanistan OR albania OR algeria OR american samoa OR angola OR "antigua and barbuda" OR antigua OR barbuda OR argentina OR armenia OR armenian OR aruba OR azerbaijan OR bahrain OR bangladesh OR barbados OR republic of belarus OR belarus OR byelarus OR belorussia OR byelorussian OR belize OR british honduras OR benin OR dahomey OR bhutan OR bolivia OR "bosnia and herzegovina" OR bosnia OR herzegovina OR botswana OR bechuanaland OR brazil OR brasil OR bulgaria OR burkina faso OR burkina fasso OR upper volta OR burundi OR urundi OR cabo verde OR cape verde OR cambodia OR kampuchea OR khmer republic OR cameroon OR cameron OR cameroun OR central african republic OR ubangi shari OR chad OR chile OR china OR colombia OR comoros OR comoro islands OR iles comores OR mayotte OR democratic republic of the congo OR democratic republic congo OR congo OR zaire OR costa rica OR "cote d'ivoire" OR "cote d' ivoire" OR cote divoire OR cote d ivoire OR ivory coast OR croatia OR cuba OR cyprus OR czech republic OR czechoslovakia OR djibouti OR french somaliland OR dominica OR dominican republic OR ecuador OR egypt OR united arab republic OR el salvador OR equatorial guinea OR spanish guinea OR eritrea OR estonia OR eswatini OR swaziland OR ethiopia OR fiji OR gabon OR gabonese republic OR gambia OR "georgia (republic)" OR georgian OR ghana OR gold coast OR gibraltar OR greece OR grenada OR guam OR guatemala OR guinea OR guinea bissau OR guyana OR british guiana OR haiti OR hispaniola OR honduras OR hungary OR india OR indonesia OR timor OR iran OR iraq OR isle of man OR jamaica OR jordan OR kazakhstan OR kazakh OR kenya OR "democratic people's republic of korea" OR republic of korea OR north korea OR south korea OR korea OR kosovo OR kyrgyzstan OR kirghizia OR kirgizstan OR kyrgyz republic OR kirghiz OR laos OR lao pdr OR "lao people's democratic republic" OR latvia OR lebanon OR lebanese republic OR lesotho OR basutoland OR liberia OR libya OR libyan arab jamahiriya OR lithuania OR macau OR macao OR "macedonia (republic)" OR macedonia OR madagascar OR malagasy republic OR malawi OR nyasaland OR malaysia OR malay federation OR malaya federation OR maldives OR indian ocean islands OR indian ocean OR mali OR malta OR micronesia OR federated states of micronesia OR kiribati OR marshall islands OR nauru OR northern mariana islands OR palau OR tuvalu OR mauritania OR mauritius OR mexico OR moldova OR moldovian OR mongolia OR montenegro OR "montenegro (republic)" OR morocco OR ifni OR mozambique OR portuguese east africa OR myanmar OR burma OR namibia OR nepal OR netherlands antilles OR nicaragua OR niger OR nigeria OR oman OR muscat OR pakistan OR panama OR papua new guinea OR new guinea OR paraguay OR peru OR philippines OR philipines OR phillipines OR phillippines OR poland OR "polish people's republic" OR portugal OR portuguese republic OR puerto rico OR romania OR russia OR russian federation OR ussr OR soviet union OR union of soviet socialist republics OR rwanda OR ruanda OR samoa OR pacific islands OR polynesia OR samoan islands OR navigator island OR navigator islands OR "sao tome and principe" OR saudi arabia OR senegal OR serbia OR seychelles OR sierra leone OR slovakia OR slovak republic OR slovenia OR melanesia OR solomon island OR solomon islands OR norfolk island OR norfolk islands OR somalia OR south africa OR south sudan OR sri lanka OR ceylon OR "saint kitts and nevis" OR "st. kitts and nevis" OR saint lucia OR "st. lucia" OR "saint vincent and the grenadines" OR saint vincent OR "st. vincent" OR grenadines OR sudan OR	982	0

		suriname OR surinam OR dutch guiana OR netherlands guiana OR syria OR syrian arab republic OR tajikistan OR tadjikistan OR tadzhikistan OR tadzhik OR tanzania OR tanganyika OR thailand OR siam OR timor leste OR east timor OR togo OR togolese republic OR tonga OR "trinidad and tobago" OR trinidad OR tobago OR tunisia OR turkey OR "turkey (republic)" OR turkmenistan OR turkmen OR uganda OR ukraine OR uruguay OR uzbekistan OR uzbek OR vanuatu OR new hebrides OR venezuela OR vietnam OR viet nam OR middle east OR west bank OR gaza OR palestine OR yemen OR yugoslavia OR zambia OR zimbabwe OR northern rhodesia OR global south OR africa south of the sahara OR "sub saharan africa" OR subsaharan africa OR africa, central OR central africa OR africa, northern OR north africa OR northern africa OR magreb OR maghrib OR sahara OR africa, southern OR southern africa OR africa, eastern OR east africa OR eastern africa OR africa, western OR west africa OR western africa OR west indies OR indian ocean islands OR caribbean region OR caribbean islands OR caribbean OR central america OR latin america OR "south and central america" OR south america OR asia, central OR central asia OR asia, northern OR north asia OR northern asia OR asia, southeastern OR southeastern asia OR south eastern asia OR southeast asia OR south east asia OR asia, western OR western asia OR europe, eastern OR east europe OR eastern europe OR developing country OR developing countries OR developing nation? OR developing population? OR developing world OR less developed countr* OR less developed nation? OR less developed population? OR less developed world OR lesser developed countr* OR lesser developed nation? OR lesser developed population? OR lesser developed world OR under developed countr* OR under developed nation? OR under developed population? OR under developed world OR underdeveloped countr* OR underdeveloped nation? OR underdeveloped population? OR underdeveloped world OR middle income countr* OR middle income nation? OR middle income population? OR low income countr* OR low income nation? OR low income population? OR lower income countr* OR lower income nation? OR lower income population? OR underserved countr* OR underserved nation? OR underserved population? OR underserved world OR under served countr* OR under served nation? OR under served population? OR under served world OR deprived countr* OR deprived nation? OR deprived population? OR deprived world OR poor countr* OR poor nation? OR poor population? OR poor world OR poorer countr* OR poorer nation? OR poorer population? OR poorer world OR developing econom* OR less developed econom* OR lesser developed econom* OR under developed econom* OR underdeveloped econom* OR middle income econom* OR low income econom* OR lower income econom* OR low gdp OR low gnp OR low gross domestic OR low gross national OR lower gdp OR lower gnp OR lower gross domestic OR lower gross national OR lmic OR lmics OR third world OR lami countr* OR transitional countr* OR emerging economies OR emerging nation?)<br>3. noft(afghan OR afghans OR afghani OR albanian? algerian? OR american samoan? OR angolan? OR antiguan? OR barbudan? OR argentine? OR argentinian? OR argentinean? OR armenian? OR aruban? OR azerbaijani? OR bahraini? OR bangladeshi? OR bangalees OR bajan? OR belarusian? OR byelorussian? OR belizean? OR beninese? OR bhutanese OR bolivian? OR bosnian? OR botswana OR batswana OR brazilian? OR brasilian? OR bulgarian? OR burkinabe OR burkinese OR burundian? OR cape verdean? OR cabo verdean? OR cambodian? OR khmer OR cameroonian? OR central african? OR chadian? OR chilean? OR chinese OR colombian? OR comorian? OR congolese OR costa rican? OR ivorian? OR croatian? OR cuban? OR cypriot? OR czech? OR djiboutian? OR dominican? OR ecuadorian? OR egyptian? OR salvadoran? OR equatorial guinean? OR equatoguinean? OR eritrean? OR estonian? OR swazi? OR swati? OR ethiopian? OR fijian OR gabonese OR gabonaise OR gambian? OR georgian? OR ghanaian? OR gibraltarian? OR greek? OR grenadian? OR guamanian? OR guatemalan? OR guinean? OR bissau guinean? OR guyanese OR haitian? OR honduran? OR hungarian? OR indian? OR indonesian? OR iranian? OR iraqian? OR iraqi? OR manx OR jamaican? OR jordanian? OR kazakhstani? OR kenyan? OR kirabati OR kirabatian? OR north korean? OR korean? OR kosovar? OR kosovan? OR kyrgyz* OR lao OR laotian? OR latvian? OR lebanese OR lesothan? OR lesothonian? OR mosotho OR basotho OR liberian? OR libyan? OR lithuanian? OR macanese OR macedonian? OR malagasy OR madagascan? OR malawian? OR malaysian? OR maldivian? OR malian? OR maltese OR marshallese? OR mauritanian? OR mauritian? OR mexican? OR micronesian? OR moldovan? OR mongolian? OR mongol OR montenegrin? OR moroccan? OR mozambican? OR burmese OR myanma OR namibian? OR nauruan? OR nepali OR nepalese OR netherlands antillean? OR nicaraguan? OR nigerien? OR nigerian? OR northern mariana islander? OR mariana? OR omani? OR pakistani? OR palauan? OR panamanian? OR papua new guinean? OR paraguayan? OR peruvian? OR philippine? OR philipine? OR phillipine? OR phillippine? OR filipino? OR filipina? OR polish OR pole OR poles OR portuguese OR puerto rican? OR romanian? OR russian? OR soviet people OR soviet population OR rwandan? OR rwandese OR ruandan? OR ruandese OR samoan? OR sao tomean? OR santomean? OR saudi arabian? OR saudi? OR senegalese OR serbian? OR montenegrin? OR seychellois OR seychelloise? OR sierra leonean? OR slovak? OR slovene? OR solomon islander? OR somali? OR south african? OR south sudanese OR sri lankan? OR ceylonese OR kittitian? OR nevisian? OR saint lucian? OR vincentian? OR sudanese OR surinamese? OR syrian? OR tajik? OR tajikistani? OR tanzanian? OR tanganyikan? OR thai OR timorese? OR togolese OR tongan? OR trinidadian? OR tobagonian? OR tunisian? OR turk? OR turkish OR turkmen? OR tuvaluan? OR ugandan? OR ukrainian? OR uruguayan? OR uzbek? OR vanuatu* OR venezuelan? OR vietnamese OR yemeni? OR yemenite? OR yemenese OR yugoslav? OR yugoslavian? OR zambian? OR zimbabwean?))<br>4. noft("mental health" OR "mental illness*" OR "mental disorder*" OR "psychological disorder*" OR "psychiatric disorder*" OR psychopatholog* OR anxiet* OR depress* OR "post-traumatic stress" OR "posttraumatic stress" OR PTSD OR suicide OR "self harm"		

		OR "substance use" OR "substance abuse" OR "alcohol use" OR "alcohol abuse" OR "drug use" OR "drug abuse" OR "mood disorder*"))<br>5. 2 OR 3<br>6. 1 AND 4 AND 5<br>7. 6 pd(20070101-20191231)		

[a]English language limit used to increase relevance of first 100 results (database contained a substantial proportion of non-English records).

Table S9: Grey literature search strategy for search engine searching.

Date	Search Engine and URL	# Search Results	# Refs Identified
13-Sep-20	Google (https://www.google.ca/)	~59,600,000	0
13-Sep-20	DuckDuckGo (https://duckduckgo.com/)	Not reported	0

Notes: The following search strategy was used: (climate change OR natural disaster OR weather OR pollution) AND (anxiety OR depression OR stress OR suicide OR substance abuse). No country-specific terms were included in the search due to length.

Table S10: Information collected by the data extraction form.

**STUDY IDENTIFICATION**	Study ID (Author-Year)<br>Citation (NLM format)<br>Source type (e.g., journal, report, conference abstract)
**STUDY DESIGN**	Study type (i.e., qualitative, quantitative, mixed methods)<br>Study design (e.g., RCT, prospective cohort, cross-sectional)<br>Study time frame (i.e., length of follow-up, timeframe of study, timeframe of data)<br>Country<br>Scope (i.e., local, regional, national, international)<br>Study population (brief description)
**CLIMATE CHANGE EXPOSURE**	Description of exposure<br>Classification of climate change exposure (e.g., temperature, precipitation, natural disaster)<br>Classification of climate-related disaster (if applicable, according to EM-DAT)<br>Description of reference group (if applicable)<br>Exposure measurement (tools used, validation methods)
**MENTAL HEALTH OUTCOME**	Description of outcome<br>Classification of mental health outcome (type of mental illness)<br>Outcome measurement (tools used, validation methods)<br>Nature of relationship between climate change and mental health (i.e., direct, indirect, neither, not specified)
**BASELINE CHARACTERISTICS**	Sample size<br>Sample characteristics (e.g., age, gender)
**RESULTS**	Analysis strategy (brief description)<br>Key quantitative findings<br>Measures of effect<br>Key qualitative findings
**OTHER**	Notes

References

1. Brooks N, Adger WN. Country level risk measures of climate-related natural disasters and implications for adaptation to climate change [Internet]. Norwich, UK: Tyndall Centre for Climate Change Research; 2003 Jan. Report No.: Working Paper 26. Available from: https://pdfs.semanticscholar.org/7b5f/da4b0d1a94426bcdbfb2c75df2bcbfc481aa.pdf

2. Daoud A, Halleröd B, Guha-Sapir D. What is the association between absolute child poverty, poor governance, and natural disasters? A global comparison of some of the realities of climate change. PLOS ONE. 2016 Apr 14;11(4):e0153296.
